# First histological observations on the incorporation of a novel nanocrystalline hydroxyapatite paste OSTIM^® ^in human cancellous bone

**DOI:** 10.1186/1471-2474-7-50

**Published:** 2006-06-08

**Authors:** Franz-Xaver Huber, Orlin Belyaev, Joachim Hillmeier, Hans-Juergen Kock, Colette Huber, Peter-Juergen Meeder, Irina Berger

**Affiliations:** 1 Surgical Clinic, Division of Traumatology and Reconstructive Surgery, University of Heidelberg, INF 110, 69120 Heidelberg, Germany.; 2 Department of General Pathology, University of Heidelberg, INF 220/221, 69120 Heidelberg, Germany

## Abstract

**Background:**

A commercially available nanocrystalline hydroxyapatite paste Ostim^® ^has been reported in few recent studies to surpass other synthetic bone substitutes with respect to the observed clinical results. However, the integration of this implantable material has been histologically evaluated only in animal experimental models up to now. This study aimed to evaluate the tissue incorporation of Ostim^® ^in human cancellous bone after reconstructive bone surgery for trauma.

**Methods:**

Biopsy specimens from 6 adult patients with a total of 7 tibial, calcaneal or distal radial fractures were obtained at the time of osteosynthesis removal. The median interval from initial operation to tissue sampling was 13 (range 3–15) months. Samples were stained with Masson-Goldner, von Kossa, and toluidine blue. Osteoid volume, trabecular width and bone volume, and cortical porosity were analyzed. Samples were immunolabeled with antibodies against CD68, CD56 and human prolyl 4-hydroxylase to detect macrophages, osteoblasts, and fibroblasts, respectively. TRAP stainings were used to identify osteoclasts.

**Results:**

Histomorphometric data indicated good regeneration with normal bone turnover: mean osteoid volume was 1.93% of the trabecular bone mass, trabecular bone volume – 28.4%, trabecular width – 225.12 μm, and porosity index – 2.6%. Cortical and spongious bone tissue were well structured. Neither inflammatory reaction, nor osteofibrosis or osteonecrosis were observed. The implanted material was widely absorbed.

**Conclusion:**

The studied nanocrystalline hydroxyapatite paste showed good tissue incorporation. It is highly biocompatible and appears to be a suitable bone substitute for juxtaarticular comminuted fractures in combination with a stable screw-plate osteosynthesis.

## Background

Operative reconstruction of bone defects beyond a certain size is mandatory and still remains a challenge to trauma and orthopedic surgeons. Every year, millions of people are suffering from bone defects arising from trauma, tumor or bone diseases [1]. In about 10% of all reconstructive operations because of traumatic, resectional, or congenital defects, bone transplants and bone substitute materials are necessary [2].

Autologous cancellous bone remains the "gold standard" in the reconstruction of bone defects because of its unsurpassed biological activity even in implant sites with low osteogenic potential. The main disadvantages of human autografts are their limited quantity and the morbidity associated with their harvest [3,4]. Human allografts offer an abundantly available alternative, which circumvents the potential morbidity of autograft harvest, but relies on a sophisticated bone banking system and carries the potential of disease transmission and immunogenicity. Furthermore, the structural, mechanical, and resorption properties of allografts are usually much altered by processing, preservation, and sterilization techniques [5]. The relative concerns over the use of either autograft or allograft have led to the development of numerous entirely synthetic bone graft substitutes.

Mineral bone substitutes have been in widespread clinical use for a number of years. The most commonly used mineral substitutes for bone defect and trauma applications as implant coatings and defect fillers are thermally processed hydroxyapatite (HA) and tricalcium phosphate. Due to their osteoconductive potential and the absence of antigenicity, osteointegration usually occurs quickly and reliably in sterile and well vascularized sites [6]. Hydroxyapatite has a similar crystal structure to that of bone mineral and has been investigated as a bone replacement material for over 30 years [7,8].

**Figure 1:**
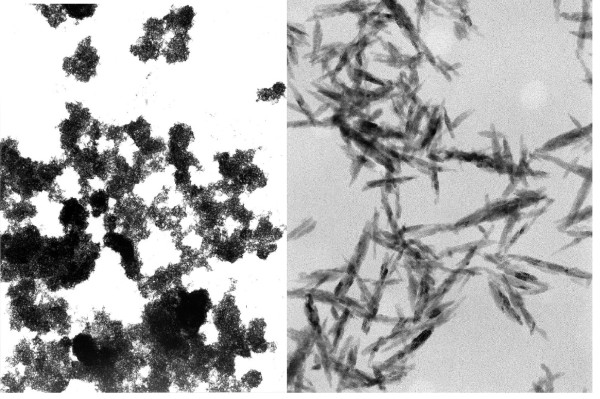
Pure nanocrystalline hydroxyapatite (Ostim^®^) forms aggregates of needle-shaped crystals in transmission electron microscopy (magnification: left 7500×; right 150 000×)

The HA used in the present study is a fully synthetic and fully resorbable injectable nanocrystalline paste [Ca_10_(PO_4_)_6_(OH)_2_] (Ostim^®^, Osartis, Obernburg, Germany), and consists of a suspension of pure HA in water prepared by a wet chemical reaction. The needle shaped HA crystals form agglomerates in transmission electron microscopy (Fig. 1). The average crystallite size is 100 nm/20 nm/3 nm, the atomic ratio of calcium-phosphorus is 1.67. Ostim^® ^paste does not harden after application into the bone and is free of endothermical heating. It is characterized by a large bioactive specific surface area of 106 m^2^/g [9]. The Ostim^® ^syringe in the double sterile pack can be used to apply paste to the bone defect directly or by means of applicators. Although Ostim^® ^has already been successfully used in parodontal surgery [10,11] and is approved for clinical use as bone substitute, no histological observations in humans have been reported so far.

The current study presents the first histological observations on the incorporation of this nanocrystalline HA paste in human cancellous bone in a group of patients, treated for various types of bone fractures.

## Methods

Ostim^® ^was used to fill bone defects in human patients, prospectively recruited for the treatment of traumatic bone defects of radius, calcaneus and tibia. Between June 2004 and October 2005 seven biopsies were performed in six patients at the time of removal of their fracture fixation devices (Table 1). The study was conducted in accordance with the Declaration of Helsinki (1996 revision). The ethics committee of the University of Heidelberg approved the study. Specific informed consent for participation in the research was obtained from all patients.

**Table 1:** Characteristics of patients from whom bone biopsies were obtained.

No	Sex	Age (years)*	Location of fracture	Interval to biopsy (mo)
1	F	31	Tibial head	14
2	M	31	Tibial head	13
3	M	54	Calcaneus	15
4	M	44	Calcaneus	3
5	M	44	Distal radius	13
6	F	64	Distal radius right Distal radius left	12 12

		44 (median)		13 (median)

* age at the time of biopsy

### Reconstruction of distal radial defects

**Figure 2:**
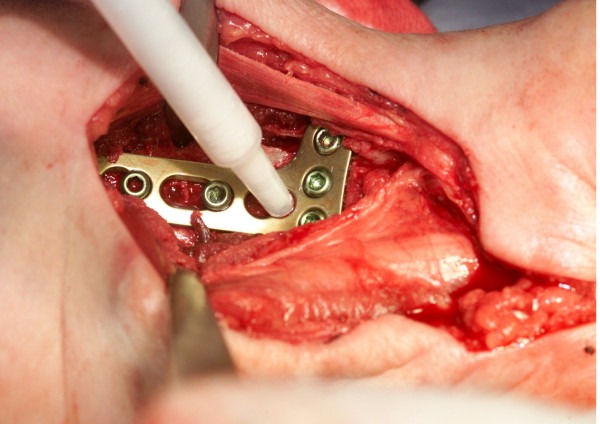
An angularly stable screw-plate system is used for fixation. The metaphyseal defect of the radius is filled with a nanocrystalline hydroxyapatite paste through a free screw-hole in the metal plate.

Two patients had a total of three fractures of the distal radius. In the female patient with bilateral radius fractures, biopsies were taken from both sides during synchronous plate removal. A standard palmar approach followed by a carpal canal dissection was used in all cases. The correct radius length was achieved by reduction with the use of a sterile hand extension set. The correct reduction and metal plate position were radiologically confirmed. Cases at a high risk of perioperative redislocation, were additionally treated with the use of temporary 1.6 mm Kirschner-wires. All K-wires and none angularly stable screws used as repositioning aids were removed and replaced with LCP-screws before the end of the operation. The final step of the operation following the definitive fixation of the plate (angular stable 3.5 mm titanium screw-plate system made by Synthes/Oberndorf-Switzerland), was the void filling with Ostim^®^. The median quantity used was 2 ml. The paste was inserted from the palmar side into the metaphyseal defect area (Fig. 2).

### Reconstruction of calcaneal defects

An extended lateral approach with an L-shaped incision was performed, beginning proximally, over the lateral aspect of the heel. It was then continued distally between the lateral malleolus and the posterior and inferior border of the heel. The peroneal tendons were then mobilized within their sheath and held back. After exposing the lateral calcaneal surface, which allowed the identification of the fracture lines, anatomic reduction was performed. In most cases we began with the reduction of the tuberosity fragment with the aid of a Schanz screw inserted dorsally. Further anatomical reduction was also conducted using various reduction aids, e.g. provisional K-wires. Special attention was paid to the exact reconstruction of the posterior facet. After realigning the impacted area, defect zones of various volumes normally remained behind. At that time-point the need of HA defect filling was definitively determined. The defect was then filled with a median volume of 5 ml Ostim^® ^after partial fixation of the bone using a calcaneal plate (Calcaneus Conventional^® ^100% titanium plate – Litos Company, Hamburg, Germany). The osteoreconstructive result was examined intraoperatively by diaphanoscopy.

### Reconstruction of tibial head defects

A decision about the type of surgical access to be used (lateral, medial, posterolateral or biportal) was based upon the fracture morphology. The deep incision went straight down to the bone leaving a narrow border of fascia attached to the tibia. The impacted articular surface had to be elevated. In order to secure the achieved reposition K-wires were temporary inserted. The cavity walls were coated with the absorbable hydroxyapatite paste (Ostim^®^) before a hydroxyapatite ceramic core (Cerabone^®^) was inserted. Once the ceramic was inserted, it was then covered with more hydroxyapatite paste before closing the cortical window. The bone voids were filled with 5 ml to 10 ml Ostim^® ^(median volume – 9 ml). Repositioning results were preserved by the implantation of an angular stable titanium screw-plate system (Synthes/Oberndorf-Switzerland).

### Histological preparations

**Figure 3a:**
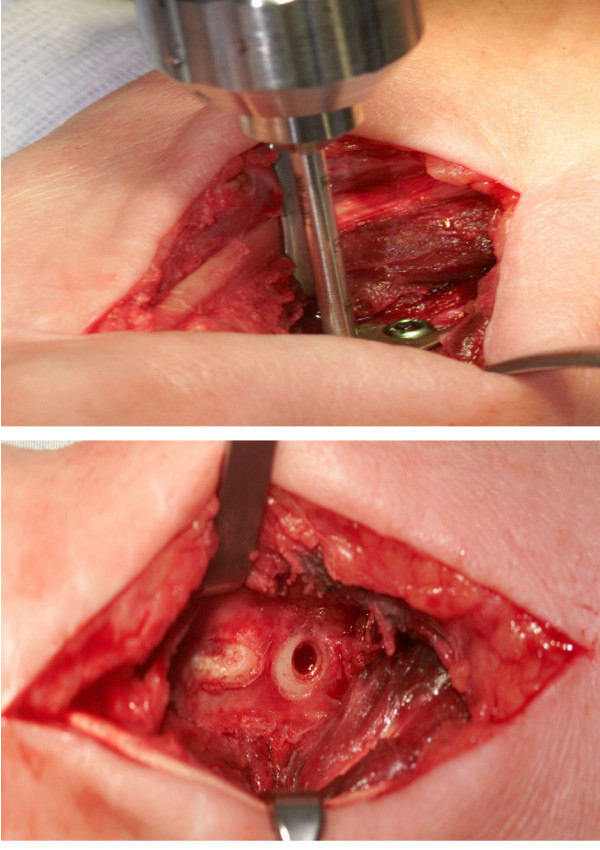
The DBCS drill (Biomet Merck, Darmstadt, Germany) is used to obtain a cylindrical biopsy. The plate is still in place at that time. **B** Intraoperative view of the radius after drilling and plate removal. The biopsy hole has the diameter of a screw hole, so stability of the reconstructed bone is threatened in no way.

Cylindrical biopsy specimens measuring 1.0–3.0 cm in length with a diameter of 5 mm, corresponding to a volume of 0.2–0.6 ml, were obtained for histological and immunohistochemical analysis with a water-cooled hollow drill (DBCS Diamond Bone Cutting System, Biomet Merck, Darmstadt, Germany) (Fig. 3a and Fig. 3b). After removal of the metal plates, the drill was positioned towards the center of the substitute material implantation site by using former screw holes, diaphanoscopy, and X-ray images for orientation. In cases of tibial head fractures biopsies were taken only from the places where Ostim^® ^had been applied. The tissue probes were fixed in 4% Formaldehyde in PBS-buffer. Using the method of Donath and Breuner 1982 [12] undecalcified bone samples were embedded in Technovit 9100^® ^New (Heraeus Kulzer, Hanau, Germany) – a low temperature methylmethacrylate embedding system that significantly improves tissue antigenicity preservation allowing polymerisation at -20°C and quantitative assessment of bone-matrix proteins. Thin 7 μm sections were stained with Masson-Goldner, von Kossa, and toluidine blue. Osteoid volume (%), trabecular width, trabecular bone volume (%), and cortical porosity (%) were analyzed. Histological examination and photography were performed using a Zeiss Axiophot microscope (Zeiss, Oberkochen, Germany). All bone samples were additionally labelled with antibodies against CD 68, CD56 and human prolyl 4-hydroxylase (h4Ph). The CD68 marker, which stains lysosomes and is therefore mainly found in phagocyting cells, was used to identify macrophages. The CD56 was used for recognizing osteoblasts. Prolyl 4-hydroxylase plays a central role in the synthesis of all collagen types and is a marker for collagen producing cells [13]. Therefore it was used for detection of fibroblasts. The following mouse anti-human antibodies were used: CD68, 1:500 (clone KP1; Dako, Germany); h4Ph, 1:400 (Medicorp, Montreal, Canada); CD56, 1:400 (Santa Cruze, Germany). For screening of h4Ph, CD 68 and CD56 expression the primary antibodies were diluted in 0.1 M Na-phosphate buffer, pH 7.0, containing 0,5% protease-free bovine serum albumin. Immunostainings were performed using the conventional alkaline phosphatase anti-alkaline phosphatase (APAAP) technique. Immunohistochemical TRAP (tartrate-resistant acid phosphatase) -staining (mouse-antihuman antibody, 1:100; Dako, Germany) was used to identify osteoclasts via ABC detection method. The sections were finally counterstained with hematoxylin and mounted. Histomorphometry was performed using point counting and linear intercept techniques.

## Results

**Figure 4:**
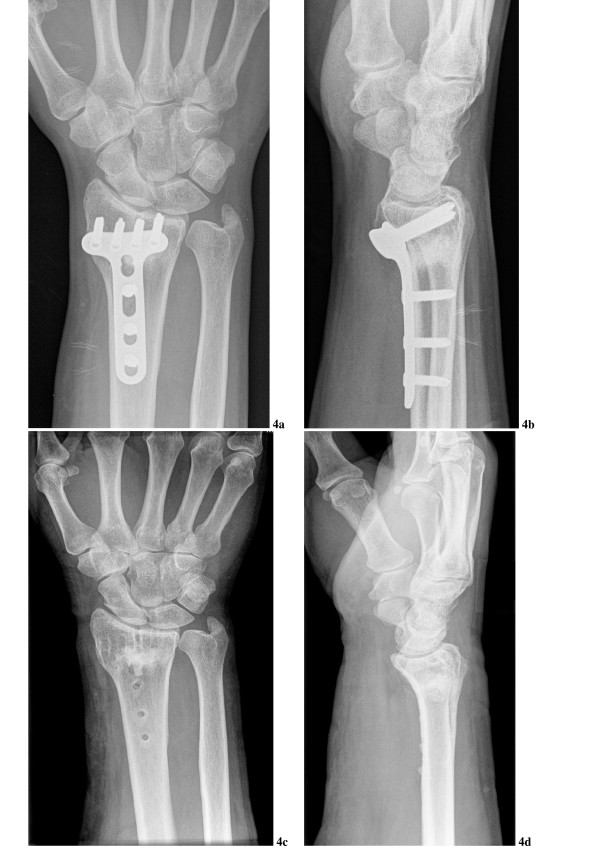
X-ray images of the hand of patient No. 5 three months after the reconstruction (**4a **and **4b**) and after the plate removal 10 months later (**4c **and **4d**). New bone has refilled the metaphyseal defect.

The postoperative course after fracture stabilization and the above described defect reconstruction was uneventful. All soft tissue wounds showed primary healing; fracture consolidation was not retarded (Fig. 4a, b, c, d). Clinical and radiological follow-up did not indicate inflammatory tissue reactions or osteolysis. Implant removal was timed according to the standards at our institution, provided that fracture consolidation had been confirmed radiologically. One patient with calcaneal fracture required early implant removal after three months because of clinically suspected local infection. However, infection could not be confirmed microbiologically and the fracture was already consolidated. The postoperative course after implant removal and biopsy was also uneventful in all patients.

**Figure 5:**
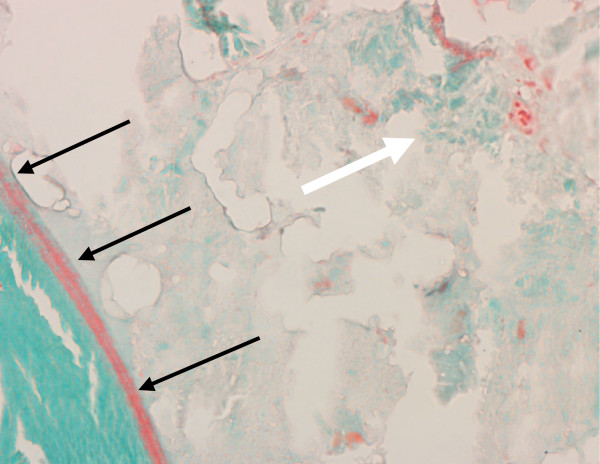
Remnants of nanocrystalline hydroxyapatite paste in the medullary space (white arrow). The border between nanocrystalline hydroxyapatite paste and newly formed cancellous bone is shown (black arrows). Histological section, Masson-Goldner staining, 40×.

**Figure 6:**
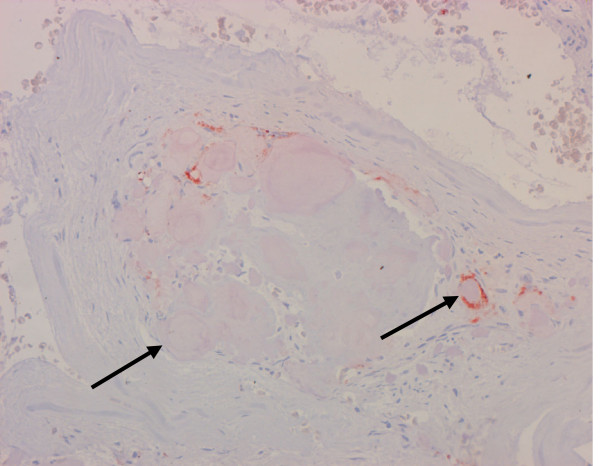
Low number of macrophages (arrows) localized in close vicinity to particles of the hydroxyapatite paste. Histological section, CD68 immunostaining.

**Figure 7:**
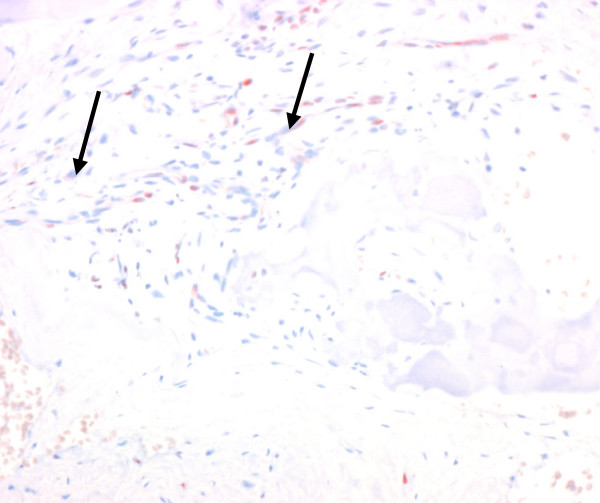
Low number of fibroblasts (arrows) distributed diffusely in the medullary space containing nanocrystalline hydroxyapatite paste. Histological section, h4Ph immunostaining, 24×.

**Figure 8:**
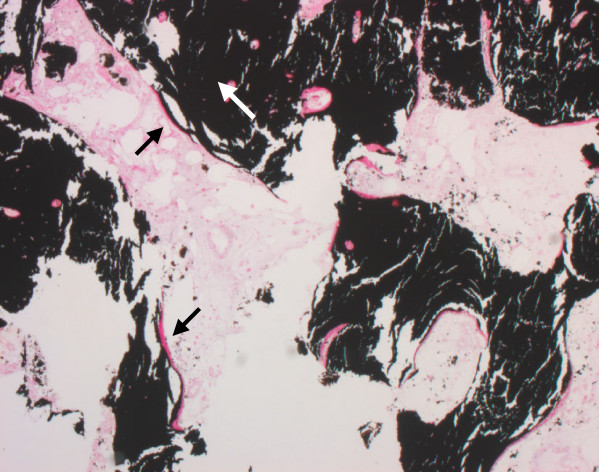
Well structured trabecular bone tissue (white arrow) with osteoid on the trabecular surface (black arrows). Histological section, von Kossa staining, 8×.

**Figure 9:**
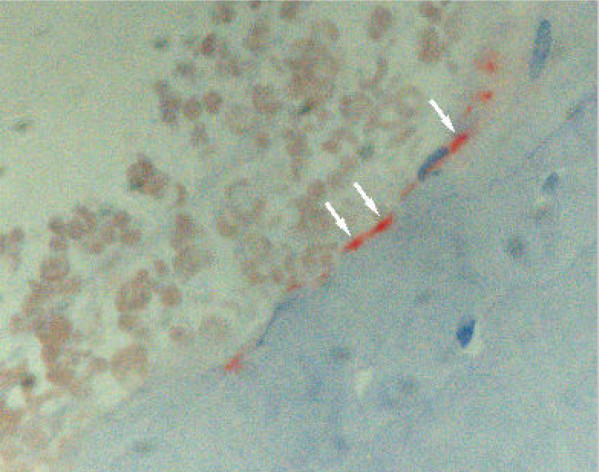
A layer of red-stained osteoblasts (white arrows) are found on the surface of newly formed trabecular bone. Histological section, CD56 immunostaining, 40×.

**Figure 10:**
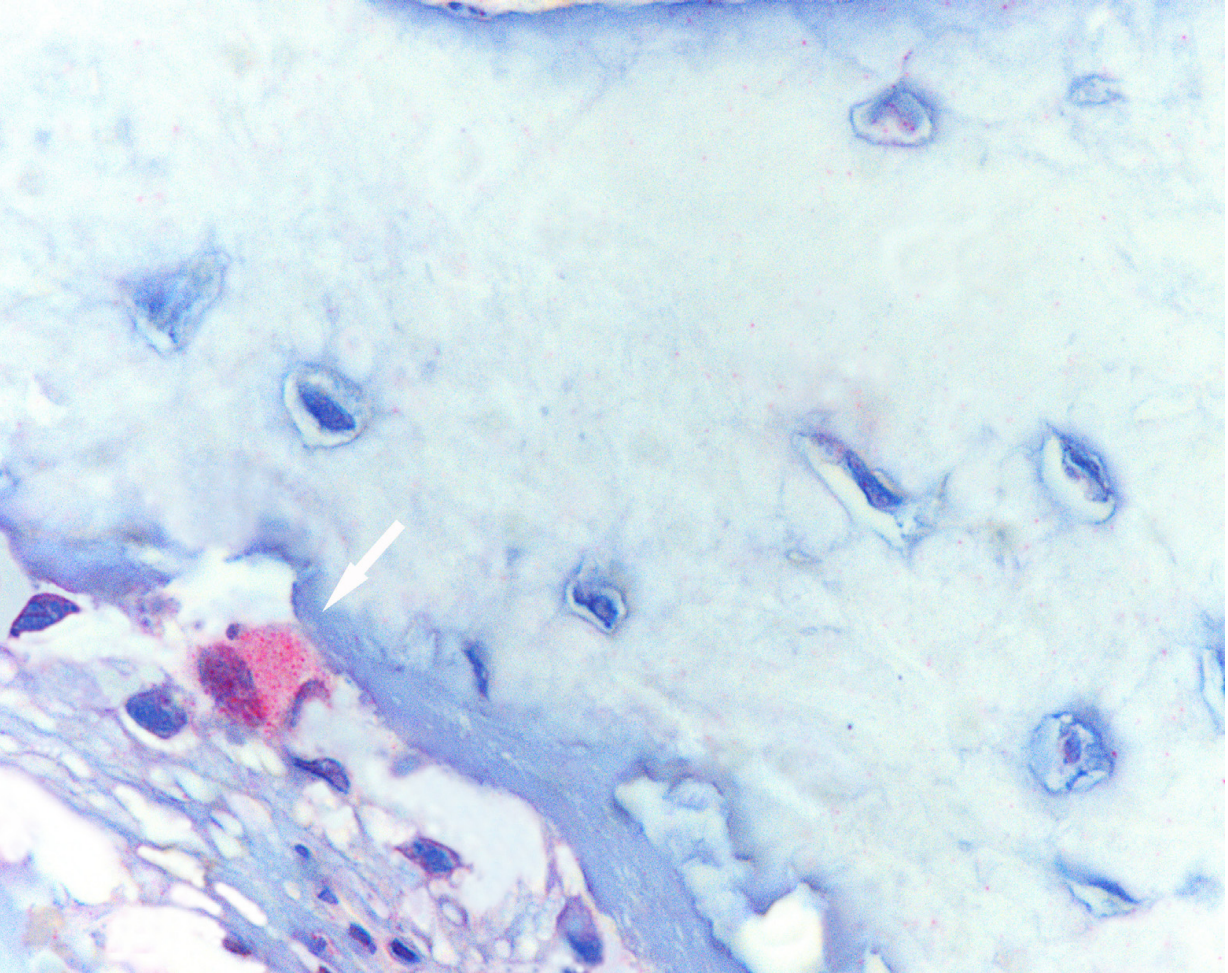
A red-stained osteoclast (white arrow) within a bone trabecle. Histological section, TRAP immunohistochemical staining, 64×.

Histological analysis of bone biopsy showed well structured cortical and cancellous bone tissue with focal fibrosis of the medullary space. All biopsy specimens contained residues of the substitute material. Within the medullary space remaining particles of amorphic HA implant material were seen in all samples (Fig. 5). In these areas low number of macrophages and fibroblasts were detected (Fig. 6, 7); the implant particles were easily seen and covered by newly formed bone. Bone healing, and ramifications of trabecular bone could be seen between the implant particles. In all specimens, new bone formation was clearly visible beginning with the deposition of osteoid directly onto the substitute material and secondary mineralization in the presence of cell layers resembling osteoblasts (Fig. 8). Osteoblasts were regularly distributed on the surface of bone trabecles (Fig. 9). The mean trabecular bone volume constituted 28,4 ± 3,82% with a mean trabecular width of 225,12 ± 16,30 μm. The osteoid volume comprised approximately 1.93 ± 0,22% of the trabecular bone mass. The osteoclastic resorptive surface was less than 0,1% of the surface of the trabecular bone. Singular osteoclasts were found within the bone trabecles (Fig. 10). The porosity index was 2,60 ± 1.33% of the bone tissue. Generally, there was an intimate contact between implant and bone, without any fibrous interface. Residues of the hydroxyapatite were connected by new trabecles, which showed a tendency to form spatial patterns reminiscent of the mechanical orientation seen in normal cancellous bone. Cells in close contact with the implant material either resembled osteoblasts or, in some sites, appeared to be resorbing the material. These latter cells contained several nuclei and were lying in shallow lacunae on the hydroxiapatite surface possibly resulting from their resorptive activity. Adjoining soft tissues did not exhibit any signs of inflammatory tissue reaction. Instead, medullary tissue consisted of differentiated fat cells and blood vessels. Cavities within the foreign material also contained adipocytes and blood vessels. While in some areas larger polynuclear cells were visible adjacent to the inner wall, in other locations a circular deposition of new bone had already occurred within these cavities.

In summary, the morphological features of the bone tissue showed good regeneration of the bone with normal bone turnover. There was neither inflammatory reaction, nor osteonecrosis. The implanted material was widely resorbed.

## Discussion

Limited availability and donor site morbidity represent major disadvantages of autografts. As many as 39% of patients may complain of chronic pain at the donor site after iliac crest harvest [3]. Furthermore, there is an added operative time and blood loss inherent in obtaining it. Other complications include arterial and nerve injury, infection, cosmetic deformity, gait disturbance, fracture, hernia, and sacroiliac joint injury [4]. Allografts are known to be immunogenic and virus transmission remains a major cause of concern. For some of these problems, synthetic HA bone substitutes have been considered a reasonable alternative. They are known to be biocompatible, osteoconductive, non-toxic, non-inflammatory, and non-immunogenic agents [14,15]. Due to their comparatively low mechanical stability, however, they should be used primarily for the reconstruction of metaphyseal bone deficiencies and less for major diaphyseal defects. Typical clinical indications, therefore, would be juxtaarticular fractures, for example, in the distal radius, proximal humerus, proximal and distal tibia, or calcaneus. They also represent an interesting alternative for reconstructing defects in the same locations resulting from the resection of benign bone tumors. The major problem with sintered HA bone substitutes (ceramics) seems to be their failure to undergo sufficient physiological remodelling. This shortcoming is attributable mainly to the heating of mineral beyond 1000°C during the sintering process. This results in the transformation of the tiny (<60 nm) primary HA crystallites into significantly larger crystals, which are difficult to break down for osteoclasts. This leads to a relatively poor bioresorbability and limiting handling characteristics [16]. While the handling characteristics were improved by the introduction of malleable HA cements, bioresorbability has remained a problem. A great variety of calcium phosphate-based injectable bone substitutes are now commercially available: Bone Source^® ^(Howmedica, USA), Norian SRS^® ^(Norian, USA), Cementek^® ^(Teknimed SA, France), Mimics^® ^(Biomet, USA), Biocement D^® ^(Biomet Merck Ltd, Germany), Biobon^® ^(Biomet Merck Ltd, Germany; in USA distributed as α-BSM™, ETEX, USA), True bone^® ^(ETEX, USA) etc. Up to now there has been accumulating evidence that osteoclasts are able to resorb these substances, though not with the same speed for the different representatives [17,18]. It will be interesting to directly compare in future studies the resorption of Ostim^® ^and the mentioned bone substitutes, and to investigate probable advantages of the combined use of Ostim^® ^with biphasic TCP/HA granules or other newly developed materials [19].

To overcome the mentioned disadvantage of ceramics and cements, a new technology was used for the production of Ostim^®^. It is synthesized by a wet chemical reaction of precipitation under permanent pH control using CaO dispersed in water under constant stirring to maintain a suspension state and H_3_PO_4 _as starting material. The amounts of both starting materials and water lead to suspension containing 5.5% of nanosized HA crystals. This suspension with a pH of 7.5 is concentrated using filtration and subsequent evaporation process leading to a high viscous paste with an HA content of 35% and a specific surface area of 106 m^2^/g [9]. It does not harden off when mixed with blood or spongiosa and its volume stability enables it to resist bleeding pressure. Simultaneously, its viscosity enables it to be applied to form-fit in close contact with the bone [20].

The above mentioned putative functional advantages of Ostim^® ^were proved in humans by two large clinical studies in the sphere of human dentofacial surgery. In the first study, Zuev et al. in 1996 found Ostim^® ^to be not inferior to bone transplant and devoid of its shortcomings in the treatment of 395 patients with periodontal defects, including mainly periodontal abscesses. Control physical examinations were performed 3, 7, and 21 days and 1, 3, and 6 months after the operation. Control X-rays were performed 6 months after surgery. Complications in the group of 200 patients treated with Ostim^® ^were only 1.5% and never involved the site of osteoplasty, whereas the complication rate was 3.6% in the group of 195 patients treated with chemically processed bone xenotransplants [10]. In the second study, Bezrukov et al. reported excellent clinical results with the use of Ostim^® ^in 49 patients who underwent cystectomy for benign cyst-tumors of the jaws. One month after the operation defects were already beginning to lose their borders, three months after the reconstruction a full bone regeneration was radiologically observed at the place of paste application, and 6 months after the manipulation mature bone tissue with a normal configuration and optical density was confirmed at X-ray films in all cases. No procedure-related complications were reported [11].

Apart from the above two trials, no other reports on the applicability of Ostim^® ^in human medicine are currently to be found in world literature. Experiments with the paste were performed in several animal studies, some of them including histological observations. It was well tolerated in dogs, sheep, rabbits and pigs. The first one of these studies, which investigated the histological integration of Ostim^® ^in the dental wall of four dogs, was reported by Grigor'ian et al. in 2000, who revealed excellent osteoinductive and osteoconductive properties of Ostim^® ^[21]. Thorwarth et al. reported 44.2–53.9% mineralized bone substance in pigs after 3–6 months in the region of the reconstructed defect and 0% remnants of the bone substitute 6 months after its application [22]. As the paste does not harden in situ, cell migration into the implantation area coincided with revascularization. Fragmentation of the paste was observed in all animal models immediately after implantation. That fragmentation into round particles of different size must be regarded as an essential step toward successful reconstitution of tissue integrity. It enables cellular infiltration of the implantation site and enables osteoblasts to perform bone formation in an osteoconductive manner. After implantation of the nanocrystalline HA paste into tibial head defects of sheep, the voids were completely filled with new bone even after 60 days. Due to proceeding bone healing, ramifications of trabecular bone could be seen between the implant particles. This spatial ingrowth pattern, taken together with the stimulation of osteoblast activity, supports complete reconstitution (e.g. bridging) of critical-size defects of animals within 4 weeks [2].

Although our study reports on a series of biopsy specimens taken at different time intervals from human patients with various types of fractures rather than from laboratory animals with standardized defects, the basic pattern of integration of the nanocrystalline hydroxyapatite appears to be the same in animals and humans: there is no fibrous interface, but instead an intimate contact between new bone and substitute material is established. At an early stage, a disintegration of the substitute material occurs and new bone starts to form trabeculae and osteonal structures like in conventional fracture healing.

Our findings, arising from the treatment of comminuted juxtaarticular fractures confirmed the good clinical results from the application of the nanocrystalline HA paste in dentofacial surgery. Moreover, the first histological observations on the integration of Ostim^® ^made in our small group of patients perfectly agree with the previous histological findings in experimental animal models.

## Conclusion

The nanocrystalline HA paste is easy to prepare and easy to apply by syringe in the operating room. A second surgical procedure to harvest autologous bone is not necessary, and complications at the donor site are therefore avoided. There is no risk of transmitting any disease. This safe and osteoconductive HA paste appears suitable for filling bone defects and bone cavities, showing resorption and a rapid osseous integration. Clinical results were comparable to those obtained in patients treated with autograft implants, and histology revealed that fracture healing went undisturbed and active resorption was performed by osteoclasts. This material appears to be mechanically and biologically efficient and safe. It is able to preserve well its volume, but not its form, so for the purposes of fracture reconstructions it should be always used in combination with a stable screw-plate osteosynthesis.

## Competing interests

The author(s) declare that they have no competing interests.

## Authors' contributions

FXH conceived the study, participated in acquisition of data, drafting and revising the manuscript. OB was involved in drafting and final revisions of the manuscript, participated in data analysis and interpretation. JH contributed to the design and conception, revised the manuscript, and was involved in the acquisition of data. HJK coordinated the study, participated in acquisition of material, analysis of data and helped to draft the manuscript. CH was involved in coordination of work and histological preparations, interpretation of data. PJM collected histological material for the study, revised the manuscript, coordinated the work. IB made histological preparations, analyzed them and made interpretations of the results, revised the manuscript. All authors read and approved the final manuscript.

## Pre-publication history

The pre-publication history for this paper can be accessed here:


